# A Detection Method of Pine Wilt Disease Based on Improved YOLOv11 With UAV Remote Sensing Images

**DOI:** 10.1002/ece3.72823

**Published:** 2025-12-28

**Authors:** Hua Shi, Zhenhui Zhu, Xiaozhou Feng, Yufen Xie, Hui Guo, Pengxiang Xue, Yonghang Wang

**Affiliations:** ^1^ College of Sciences, Xi'an Technological University Xi'an China; ^2^ Shaanxi Academy of Forestry Xi'an China

**Keywords:** CAA, ODConv, PWD, UAV remote sensing, YOLOv11

## Abstract

Pine wilt disease (PWD), also known as pine wilt nematode disease, is a severe forest disease caused by the pine wood nematode (*Bursaphelenchus xylophilus*), which spreads rapidly and causes severe ecological damage in China. However, the existing detection methods have low accuracy in identifying early‐stage infected trees and are prone to missing detections. To address these issues, this study proposes YOLOv11‐OC, a detection method of pine wilt disease based on improved YOLOv11 with UAV remote sensing images. The omni‐dimensional dynamic convolution (ODConv) is employed to optimize the C3K2 module, thereby enhancing the feature extraction ability for small targets and improving the accuracy of identifying early‐stage disease areas. Meanwhile, the context anchor attention (CAA) mechanism is introduced to improve the C2PSA module, enhancing the model's context awareness, effectively reducing missed detections, and improving detection performance in complex backgrounds. Experimental results demonstrate that the proposed YOLOv11‐OC algorithm outperformed the original YOLOv11, achieving a precision of 94.2%, a recall of 83.1%, a F1‐score of 88.3%, and a mean average precision (mAP) of 88.9%. Compared to the original algorithm, the improved version shows increases of 2.8% in precision, 1.0% in F1‐score, and 1.4% in mAP. Furthermore, the algorithm also demonstrates good generalization ability on the public PlantDoc dataset.

## Graphical abstract

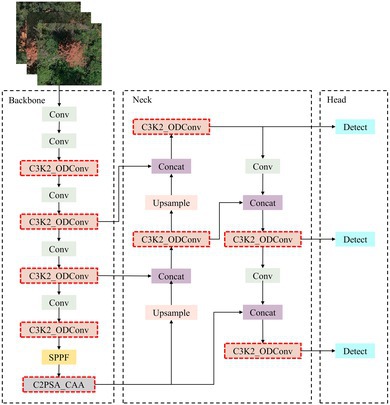

## 1 Introduction

China is a vast country with abundant forest resources and extensive forest coverage. This not only plays a vital role in ecological conservation, but also makes significant contributions to economic development, climate regulation, and biodiversity preservation (Wan et al. 2018; Zhang and Ke 2020). However, since the first introduction of the pine wood nematode into China in 1982, the country's forest resources have faced a severe threat (Wang et al. 2024; Hu et al. 2024; Wen et al. 2024). The pine wood nematode is a microscopic worm that primarily spreads through vector insects such as the pine sawyer beetle (*Monochamus alternatus*). This nematode reproduces within the xylem of pine trees, blocking the transport of water and nutrients, which leads to the wilting and death of the trees. Pine wilt disease (PWD), caused by this nematode, spreads rapidly and severely affects the health of pine trees, leading to their wilting and death, as well as large‐scale forest degradation (Hao et al. 2022; Zhou et al. 2022). This disease not only causes substantial losses in forestry production and economic benefits, but also brings about profound ecological damage. It undermines the stability of forest ecosystems, reduces their carbon sink capacity, and weakens their ability to conserve soil and water. Therefore, the scientific prevention and control of trees infected by PWD has become an urgent priority.

In recent years, unmanned aerial vehicle (UAV) remote sensing technology has developed rapidly (Luo et al. 2025; Cai et al. 2023; Li and Wang 2025; Duarte et al. 2022). Currently, the main methods for detecting trees infected by PWD include manual field surveys and UAV‐based image detection. However, manual detection primarily relies on the human eye to distinguish the color changes in diseased trees (Zhang et al. 2025; Zhou and Yang 2022), which is highly subjective and often fails to efficiently and comprehensively cover large forest areas. In contrast, UAV image detection offers significant advantages. It enables efficient data acquisition over vast regions in a short time and can accurately identify infected trees using high‐resolution imagery, greatly improving both the efficiency and accuracy of detection.

A growing number of researchers have integrated UAV remote sensing with deep learning techniques (Wu et al. 2021; Tao et al. 2020; Lim et al. 2022; Ran et al. 2024), significantly improving the accuracy of detecting trees affected by PWD. Wu and Jiang (2023) proposed a PWD detection method based on an improved mask R‐CNN and ConvNeXt. This method employs ConvNeXt for feature extraction, introduces a PA‐FPN structure, and applies normalization through the combination of group normalization and w eight standardization. Furthermore, the mask branch was optimized to enhance segmentation precision. The method demonstrated robust and accurate multi‐scale PWD detection, even in complex forest environments. Zhu et al. (2024) proposed a PWD detection approach based on an improved YOLOv7. This method integrates several attention mechanisms—SE, CEAM, ECA, and SimAM—into the YOLOv7 model, resulting in notable improvements in both precision and recall, and enabling efficient and accurate detection of trees affected by PWD. Also Yao, Song, et al. (2024) developed a method named Pine‐YOLO for PWD detection. This approach integrates dynamic snake convolution (DSConv), multidimensional collaborative attention mechanism (MCA), and Wise‐IoU v3 (WIoUv3) into the YOLOv8 network, significantly improving the accuracy of PWD detection in UAV remote sensing imagery. In another study, Su et al. (2024) proposed a detection method called PWD‐YOLOv8n. This approach integrates the coordinate attention (CA), convolutional block attention module (CBAM), bidirectional feature pyramid network (BiFPN), the lightweight FasterBlock structure, efficient multi‐scale attention (EMA), and the Inner‐SIoU loss function into the YOLOv8n framework, thereby achieving substantial improvements in detection performance. Yuan et al. (2025) proposed a detection method named YOLOv8‐RD for PWD. This model incorporates the strengths of residual learning and fuzzy deep neural networks by designing a residual fuzzy (ResFuzzy) module and a dynamic upsampling (DySample) module, which effectively filter image noise, enhance background features, and improve detection accuracy under complex interference conditions. In the same year, Ren et al. (2025) introduced a method called MASFNet for PWD tree detection. This model features a Multi‐level Attention (MLAttention) module and a spatial sampling fusion (SSF) module, which reduce false positives by expanding the receptive field and extracting fine‐grained features, thereby significantly enhancing detection accuracy. However, these studies still have limitations in certain aspects. The detection accuracy of existing methods under complex backgrounds needs to be improved, and there is still a certain rate of missed detections when dealing with issues such as branch occlusion and the complex morphology of infected trees.

Therefore, this study proposes a detection method for pine wilt disease based on the improved YOLOv11 with UAV remote sensing images. Specifically, a detection model named YOLOv11‐OC is developed by enhancing the YOLOv11 architecture with the integration of the omni‐dimensional dynamic convolution (ODConv) and the context anchor attention (CAA) modules, enabling effective detection of PWD‐infected trees. The proposed enhancements improve the model's ability to extract features from small targets (Kang 2022; Yao, Lin, et al. 2024) and long‐range diseased regions in complex environments. The ODConv module, through a multi‐dimensional attention mechanism, adaptively adjusts convolution kernel weights, thereby enhancing the model's capacity to extract features from small and complex targets. This reduces information loss and allows the model to accurately capture critical features even under challenging conditions. The CAA module improves the model's capability to capture features from long‐distance diseased regions and effectively addresses issues such as occlusion by tree branches, complex morphology of infected trees, and environmental noise, ultimately reducing missed detections. Overall, the improved YOLOv11‐OC model demonstrates higher detection accuracy and stronger environmental adaptability in the task of PWD‐infected tree detection.

## 2 Materials and Methods

### 2.1 Study Area Selection

**FIGURE 1:**
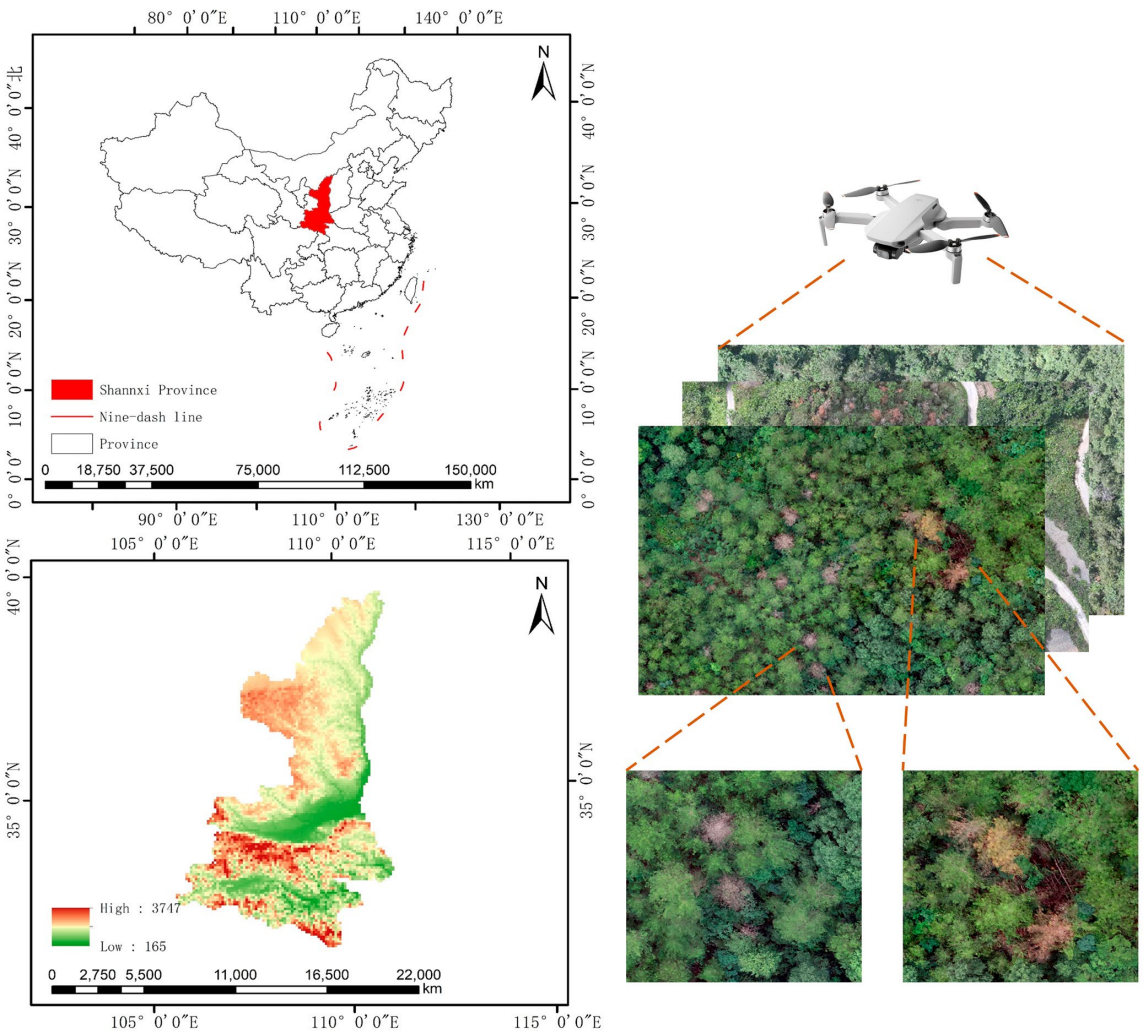
Data collection.

The study area is located in the forest regions of western China. DJI UAVs equipped with onboard cameras were used as the flight platform to collect data on pine wilt disease‐infected trees, as shown in Figure 1.

During the data acquisition process, insufficient lighting can lead to overly dark images, thereby affecting the accuracy of identifying diseased trees. To ensure adequate illumination and minimize the influence of irrelevant factors, all data images in this study were collected between 10:00 a.m. and 5:00 p.m. on clear, sunny days. Additionally, to maintain image consistency, the same UAV model was used throughout the data collection, and aerial photography was conducted at a fixed flight altitude.

### 2.2 Dataset Construction

**FIGURE 2:**
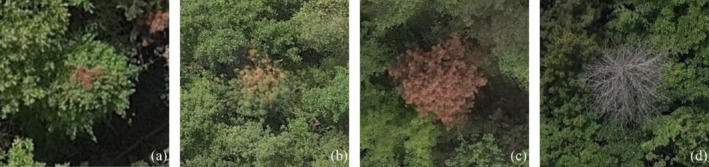
The four stages of pine wilt disease. (a) Early infection stage. (b) Disease expansion stage. (c) Severe infection stage. (d) Death stage.

In this study, PWD‐infected trees captured in UAV aerial images were selected as the research objects. The collected images were cropped into fixed‐size pixel patches to construct the dataset. In the dataset, the progression of PWD in trees is categorized into four main stages (Shi et al. 2024), as illustrated in Figure 2.

As shown in Figure 2, the progression of PWD is divided into four stages: (a) *Early infection stage*: At this stage, symptoms are not yet obvious. Slight discoloration may appear in parts of the tree crown. (b) *Disease expansion stage*: The needles and leaves of the tree gradually lose their green color, turning yellow or red. Tree growth becomes stagnant or begins to decline. (c) *Severe infection stage*: The tree exhibits a noticeably withered or reddish appearance, and the wood tissues begin to decay. (d) *Death stage*: The pine tree is completely dead, with all needles shed, bark falling off or showing visible cracks, and significant decay in the trunk wood.

**FIGURE 3:**
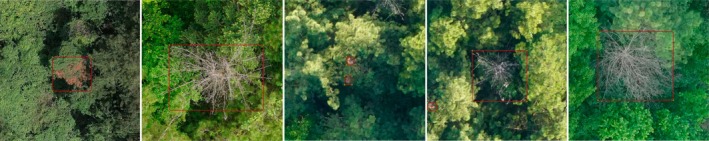
Example of dataset labeling.

To expand the dataset, data augmentation techniques such as multi‐angle rotation and brightness adjustment were applied. Based on this, under the guidance of experts from the Forestry Academy, all diseased trees in the dataset were individually annotated using a publicly available annotation tool. After the annotations were completed, a label.txt file was generated to store all the label information, facilitating subsequent model training and analysis. An example of the dataset annotation is shown in Figure 3.

The quality of the dataset directly impacts the model's learning performance and generalization ability. Ensuring that the dataset is sufficiently representative and diverse, covering different scenarios and conditions, helps the model better understand and capture key features in the data, thereby improving its performance and accuracy in real‐world applications. The specific information of the PWD‐infected tree dataset selected in this study is shown in Table 1.

**TABLE 1:** Pine wilt disease infected tree dataset.

Dataset	Number of images	Number of objects
Total	5438	14,492
Training	4138	10,673
Testing	1298	3819

### 2.3 Detecting Methods

#### 2.3.1 YOLOv11‐OC Detection Algorithm

In this study, YOLOv3, YOLOv5, YOLOv8, and YOLOv11 were first applied to detect trees in the self‐constructed dataset. The results demonstrated that YOLOv11 performed the best in detecting PWD‐infected trees. Therefore, YOLOv11 was selected as the baseline model for further research in this study.

YOLOv11, released by Ultralytics in April 2024, is an efficient object detection model featuring a three‐part architecture: Backbone, Neck, and Head. Compared to previous versions, it achieves significant improvements in both speed and efficiency. YOLOv11 introduces the C3k2 module to optimize convolutional computations and enhance feature representation. The model incorporates the C2PSA attention mechanism to effectively capture contextual information. Its Neck combines FPN and PAN structures, integrating SPPF and C2PSA modules to enhance multi‐scale feature aggregation. Overall, YOLOv11 delivers improved frame rates and average precision, making it suitable for both high‐performance computing and deployment on edge devices.

**FIGURE 4:**
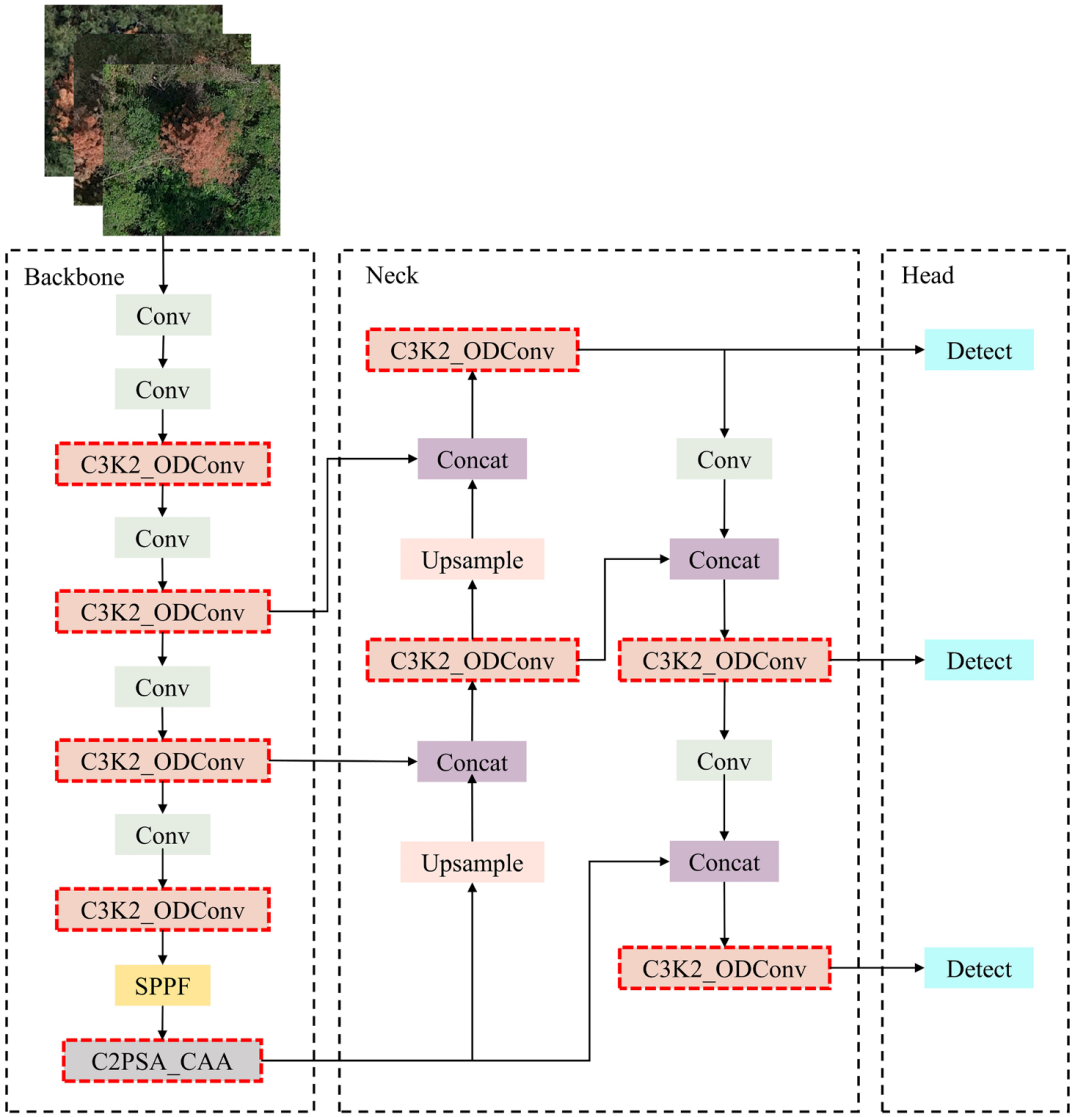
The improved YOLOv11‐OC architecture diagram.

However, several challenges arise when using YOLOv11 for detecting pine wilt disease‐infected trees. On the one hand, for small early‐stage diseased tree areas, YOLOv11 struggles to effectively extract key features. On the other hand, the convolutional modules of YOLOv11 tend to focus on local feature extraction, making it difficult to capture long‐range relational information of PWD across different tree regions, leading to missed detections. To address these challenges, this study proposes a YOLOv11‐based UAV remote sensing imagery detection method, called YOLOv11‐OC, as shown in Figure 4. The red dashed box highlights the modified module areas in this model.

The proposed YOLOv11‐OC model enhances detection performance in PWD‐infected trees in two main ways. On one hand, the omni‐dimensional dynamic convolution (ODConv) module improves the C3K2 by using a multi‐dimensional attention mechanism to adaptively adjust the convolution kernel weights, thereby enhancing the model's ability to extract features from small targets. On the other hand, the context anchor attention (CAA) module is introduced to improve the C2PSA module, boosting the model's ability to capture long‐range features of PWD‐infected trees, which reduces missed detections. These improvements further enhance detection accuracy, particularly in challenging scenarios involving branch occlusion, complex tree morphology, and environmental interference.

#### 2.3.2 Introducing the Omni‐Directional Convolution: C3K2 ODConv

To address the issue of YOLOv11's inability to effectively extract key features from small early‐stage diseased trees, the C3k2 module is improved by incorporating the omni‐dimensional dynamic convolution (ODConv) module. This enhancement uses multi‐dimensional dynamic weights to strengthen the model's ability to extract features from small targets.

The traditional C3k2 module in YOLOv11 relies solely on standard convolution, which leads to the neglect of features in small target disease areas. In this study, the omni‐dimensional dynamic convolution (ODConv) (Li et al. 2022) is adopted, which, through multi‐dimensional dynamic weight allocation, enables more precise extraction of both local and global features of PWD, thus improving detection recall. This module employs a multi‐dimensional attention mechanism and parallel strategy, focusing on four dimensions: convolution kernel dimension, spatial dimension, input channel dimension, and output channel dimension. This approach enhances the model's focus on different features, allowing for more accurate localization of complex features in defect regions, thereby improving detection accuracy. The computation formula for ODConv is as follows:

(1) $$\begin{array}{c} y=(\alpha_{w1}⊙\alpha_{f1}⊙\alpha_{c1}⊙\alpha_{s1}⊙W_{1}+\cdots \\ \;+\alpha_{\mathrm{wn}}⊙\alpha_{\mathrm{fn}}⊙\alpha_{\mathrm{cn}}⊙\alpha_{\mathrm{sn}}⊙W_{n})*x \end{array}$$

Here, *x* and *y* represent the input and output features, respectively. * Denotes the convolution operation. *W*_*i*_ represents the convolution kernel. *α*_*wi*_ indicates the attention scalar for the convolution kernel *W*_*i*_. *α*_*fi*_, *α*_*ci*_, and *α*_*si*_ are the attention scalars for the spatial dimension, input channel dimension, and output channel dimension of the convolution kernel *W*_*i*_, respectively. The operation ⊙ represents multiplication along different dimensions of the kernel space.

**FIGURE 5:**
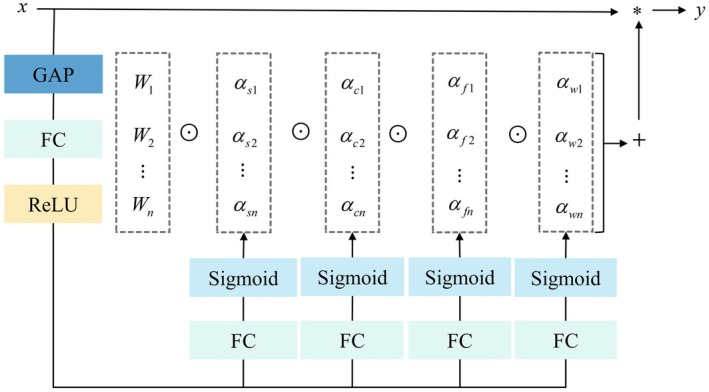
The ODConv module workflow diagram.

The workflow of the omni‐dimensional dynamic convolution (ODConv) module is illustrated in Figure 5. First, the input feature is compressed using global average pooling (GAP) to obtain a feature vector of length. This vector is then mapped to a lower‐dimensional space with a reduction ratio of *r* through a fully connected (FC) layer, in order to reduce computational complexity. Next, a ReLU activation function is applied to eliminate negative values from the features, and the resulting vector is fed into four parallel attention branches. The first three branches use FC layers followed by Sigmoid activation functions to compute attention scalars for the spatial dimension, input channels, and output channels of the convolution kernel, respectively. The fourth branch uses an FC layer followed by a Softmax activation function to compute attention scalars for the kernel number dimension. Finally, attention scalars from all four dimensions are used to perform a weighted summation with the corresponding convolution kernels. The resulting dynamic convolution kernels are then convolved with the input feature *x* to produce the output feature *y*.

#### 2.3.3 Introducing the Context Anchor Attention: C2PSA CAA

To address the issue that YOLOv11 struggles to capture long‐range contextual dependencies of PWD across different tree regions‐leading to missed detections‐this study introduces the context anchor attention (CAA) mechanism into the C2PSA module as an improvement.

In addition, this study also integrates three attention mechanisms—MLLA (mamba‐like linear attention), CGA (cascaded group attention), and CAA (context anchor attention)—into the YOLOv11 model for comparative evaluation. As shown in Table 3, the model incorporating the CAA module achieved superior performance in terms of recall and mean average precision (mAP). These results further demonstrate the effectiveness of the CAA module in enhancing the YOLOv11 model.

**FIGURE 6:**
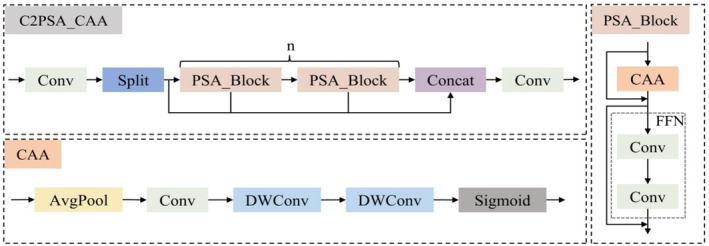
The C2PSA_CAA module architecture diagram.

The context anchor attention (CAA) mechanism (Cai et al. 2024) is a novel attention method designed to capture long‐range contextual feature information. In this study, CAA is integrated into the C2PSA module based on its characteristics to enhance the model's ability to detect diseased regions of PWD trees. The structure of the C2PSA_CAA module is illustrated in Figure 6.

The context anchor attention (CAA) module is introduced into the PSA_Block within the C2PSA structure. The architecture of the CAA module is illustrated in Figure 6. First, local region features are obtained through an average pooling layer followed by a standard convolution (Conv) operation. Then, depthwise separable convolutions (DWConv) with kernel sizes of and are applied to reduce the number of parameters while enhancing the capability for long‐range feature recognition and extraction. Subsequently, the features are integrated through another Conv operation. Finally, a Sigmoid activation function is applied to generate contextual attention features.

## 3 Experiment and Performance Analysis

### 3.1 Experimental Configuration

In the experiments, the Adam optimizer was selected to optimize the neural network. The initial learning rate was set to 0.001, with a learning rate decay factor of 0.01. During model training, the batch size was set to 4, and the number of epochs was set to 150. All experiments were conducted under the same experimental environment. Detailed experimental configurations are shown in Table 2.

**TABLE 2:** Experimental configuration.

Name	Configuration
Operating system	Windows10
CPU	Intel Core i5‐13400F
GPU	RTX 4060 Ti
GPU memory	16 GB
Python	3.11.5
Pytorch	2.0.0+cu118
CUDA version	12.6

### 3.2 Experimental Indicators

To evaluate the performance of the model in the task of pine wilt disease (PWD) tree detection, four commonly used evaluation metrics are adopted in this study: Precision (P), Recall (R), F1 score (F1), and mean average precision (mAP50). These metrics assess the detection performance of the model from different perspectives. The specific calculation formulas are as follows:

(2) $$\text{Precision}=\frac{\mathrm{TP}}{\mathrm{TP}+\mathrm{FP}}$$

(3) $$\text{Recall}=\frac{\mathrm{TP}}{\mathrm{TP}+\mathrm{FN}}$$

(4) $$F1=2\times \frac{\text{Precision}\times \text{Recall}}{\text{Precision}+\text{Recall}}$$

(5) $$\mathrm{mAP}=\frac{1}{n}\sum_{i=1}^{n}AP_{i}$$

### 3.3 Comparative Experiment of Different Attention Mechanisms

This study introduces different attention mechanisms—MLLA (mamba‐like linear attention), CGA (cascaded group attention), and CAA (context anchor attention)—into the YOLOv11 model for comparative experiments. The experimental results are shown in Table 3.

**TABLE 3:** Comparison of model training results under different attention.

Model	P%	R%	F1 score%	mAP50%	GFLOPs
YOLOv11‐MLLA	**93.6**	82.6	**87.7**	87.1	6.5
YOLOv11‐CGA	92.6	82.4	87.2	86.4	6.5
YOLOv11‐CAA	92.4	**83.3**	87.6	**88.1**	**6.4**

*Note:* Bold values indicate the positions where the corresponding indicators reach their maximum values.

In terms of model stability, the mAP50 value of the CAA module is 88.1% which is an improvement of 1.15% and 1.96% over MLLA and CGA, respectively, indicating its good stability. In terms of Recall, the CAA module achieves a Recall of 83.3%, which is an increase of 0.85% and 1.09% over MLLA and CGA, respectively, demonstrating that CAA can better identify and detect more targets, reducing the number of missed detections. Regarding computational cost, the CAA module has the lowest GFLOPs, and while improving performance, it introduces almost no additional computational burden.

**FIGURE 7:**
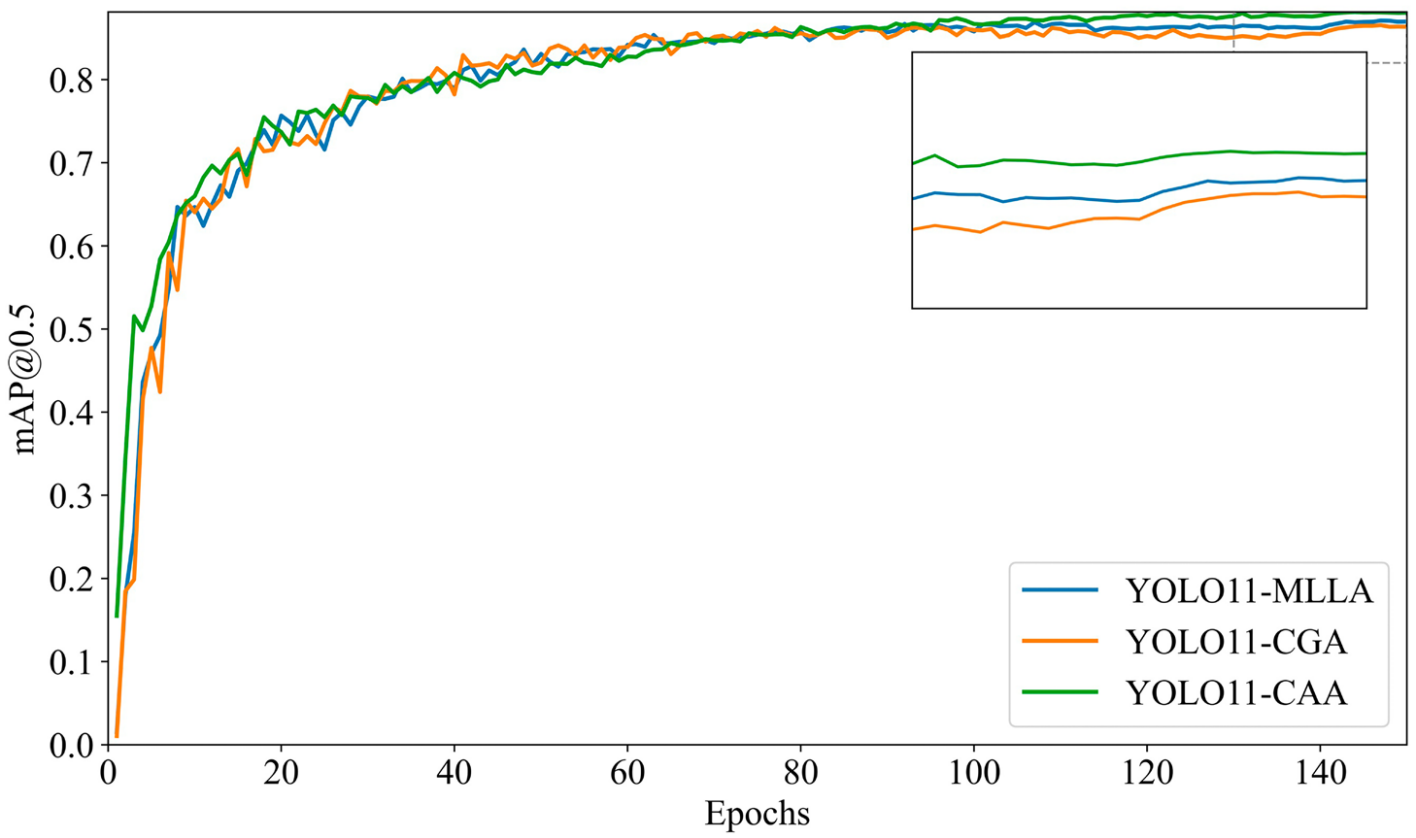
Training comparison curves of mAP@0.5 for different attention mechanisms.

The curve showing the change of mAP50 with respect to epochs for different attention mechanisms during training is plotted in Figure 7.

As shown in Figure 7, during the 150 epochs of training, the model with the CAA module demonstrates superior performance in terms of average precision. Additionally, as seen in Table 3, compared to other attention mechanisms, the CAA module achieves lower GFLOPS. This reduction in computational complexity is accompanied by a higher mAP50, maintaining excellent detection performance. Therefore, the study further improves upon the YOLOv11‐CAA model.

### 3.4 Ablation Experiments

This study improves upon the YOLOv11 model by introducing the ODConv module to optimize C3K2 and by integrating the CAA mechanism to improve C2PSA. Ablation experiments were conducted on the constructed dataset to validate the contribution of each improvement to the overall model. The results of the ablation experiments are shown in Table 4.

**TABLE 4:** Ablation study results.

Baseline model	ODConv	CAA	P%	R%	F1 score%	mAP50%	GFLOPs
YOLOv11	×	×	91.6	83.6	87.4	87.6	6.4
	✓	×	92.1	**84.6**	88.1	88.4	7.2
	×	✓	92.4	83.3	87.6	88.1	6.4
	✓	✓	**94.2**	83.1	**88.3**	**88.9**	7.2

*Note:* Bold values indicate the positions where the corresponding indicators reach their maximum values.

First, the ODConv module was introduced into the YOLOv11 model. Compared to the original YOLOv11 model, there were improvements in Precision, Recall, F1 score, and mAP50. The ODConv module requires the computation of multiple convolution kernel weights, which leads to an increase in computational complexity by 0.8, but it enhances the performance of target detection.

Next, after introducing the CAA module into the YOLOv11 model, improvements were observed in Precision, F1 score, and mAP50 compared to the original YOLOv11 model. Although there was a slight decrease in Recall, the F1 score remained at a relatively high level. This indicates that the CAA module not only enhances the model's precision and overall detection capability but also effectively balances Precision and Recall.

Finally, the proposed YOLOv11‐OC model introduces both the ODConv and CAA modules into the YOLOv11 framework. The ODConv module extracts rich dynamic features, improving the model's ability to capture early‐stage diseased tree regions. The CAA module enhances the model's contextual awareness, reducing false detections. The experimental results indicate that the YOLOv11‐OC model has achieved improvements in the metrics of Precision, F1 score, and mAP50, reaching 94.2%, 88.3%, and 88.9%, respectively. Compared with the YOLOv11 model, the Precision has increased by 2.8%, the F1 score by 1.0%, and the mAP by 1.4%. Although these increments may appear modest in numerical terms, they hold significant practical value in large‐scale forest monitoring. These enhancements can effectively improve the accuracy of detection and reduce the false‐negative rate, thereby ensuring that infected trees are promptly identified and properly treated. This, in turn, helps to effectively curb the further spread of pine wilt disease and provides robust technological support for the protection of forest resources and the sustainable development of forest ecosystems.

### 3.5 Performance Comparison of Different Methods

To validate the detection performance of the proposed model, experiments were conducted to compare YOLOv3, YOLOv5, YOLOv8, YOLOv9t, YOLOv10, YOLOv11, and the proposed YOLOv11‐OC model. The experimental results are shown in Table 5.

**TABLE 5:** Performance comparison of different models.

Model	P%	R%	F1 score%	mAP50%	GLOPs
YOLOv3	91.2	82.4	86.6	86.7	13
YOLOv5	91.3	83.2	87.0	86.7	**4.2**
YOLOv8	91.4	79.9	85.3	84.2	8.2
YOLOv9t	92.3	82.4	87.1	86.3	7.8
YOLOv10	92.4	80.6	86.1	85.7	8.4
YOLOv11	91.6	**83.6**	87.4	87.6	6.4
Proposed	**94.2**	83.1	**88.3**	**88.9**	7.2

*Note:* Bold values indicate the positions where the corresponding indicators reach their maximum values.

From the comparative experiments, it can be concluded that the proposed YOLOv11‐OC model exhibits the highest Precision among all the compared models, reaching 94.2%. Additionally, it achieves the best F1 score, which is 88.3%. Although the Recall is slightly lower than that of YOLOv5 and YOLOv11, its mAP50 value reaches 88.9%, the highest among all models, fully demonstrating its superiority in overall object detection performance. Furthermore, the YOLOv11‐OC model's computational complexity, measured in GFLOPs, is 7.2. While it is higher than YOLOv5 and YOLOv11, it is lower than both YOLOv3 and YOLOv8, indicating that the YOLOv11‐OC model achieves performance improvements while maintaining reasonable computational efficiency.

**FIGURE 8:**
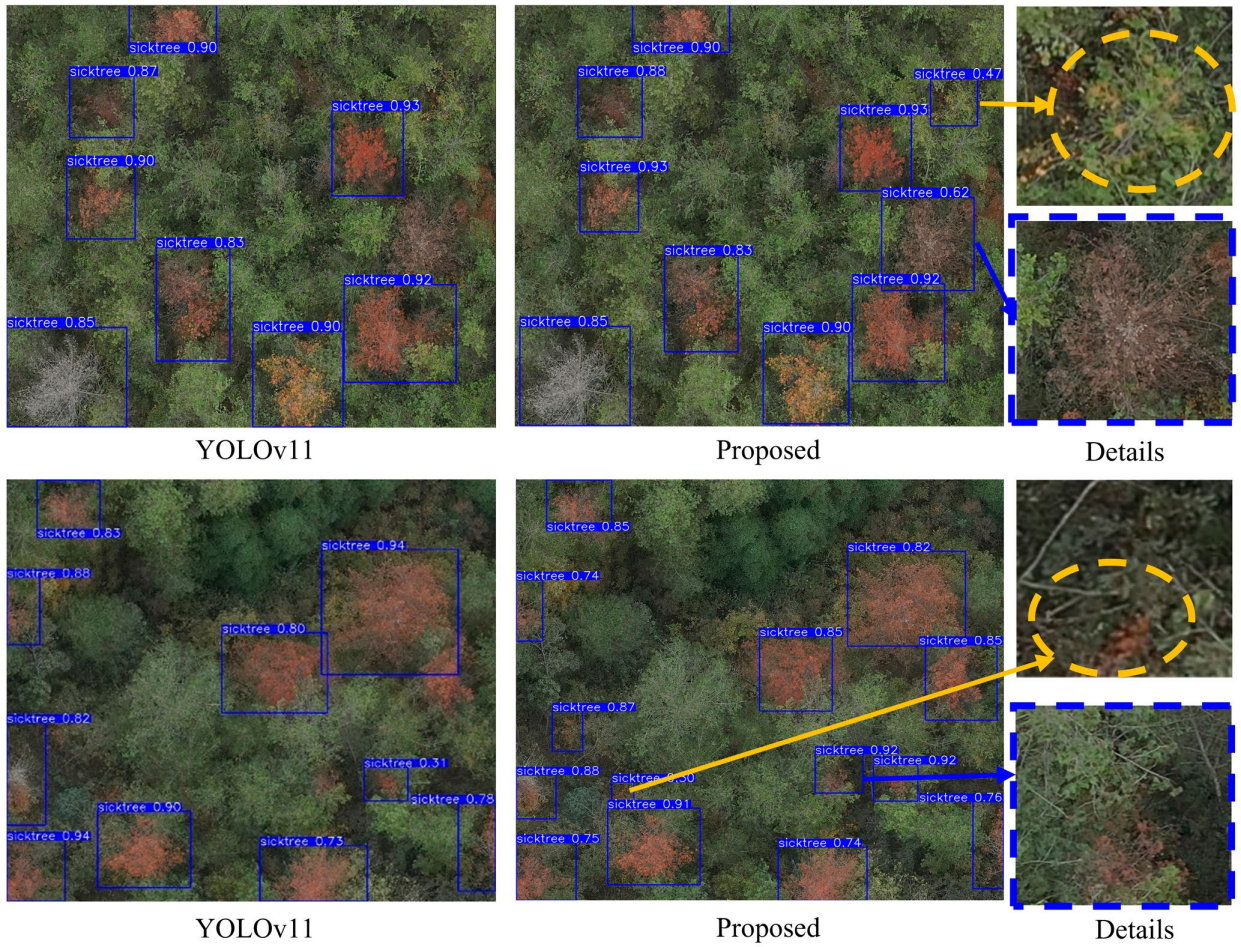
Comparison of results between YOLOv11 and YOLOv11‐OC.

To further validate the effectiveness of the improved algorithm, this paper compares the results of the YOLOv11 model with the YOLOv11‐OC model. The comparison results are shown in Figure 8.

The figure shows a comparison of detection results between the YOLOv11 model and the proposed YOLOv11‐OC model on the same image. The first column displays the detection results of the YOLOv11 model, the second column shows the detection results of the proposed YOLOv11‐OC model, and the third column presents the enlarged detail view of the detection results. In the figure, the yellow elliptical areas highlight early‐stage diseased trees detected by the YOLOv11‐OC model, while the blue dashed rectangular areas indicate diseased trees that were missed by YOLOv11 but successfully detected by YOLOv11‐OC.

As shown in Figure 8, in the detection results of the remote sensing image in the first row, the YOLOv11 model exhibits a missed detection problem for early‐stage small diseased trees in the upper right corner. By introducing the omni‐dimensional dynamic convolution (ODConv) to improve the C3K2 module of YOLOv11, the YOLOv11‐OC model enhances the detection accuracy for small targets in the yellow elliptical area. In addition, the YOLOv11‐OC model incorporates the CAA mechanism into the C2PSA module, which strengthens the model's ability to capture diseased areas of pine wilt nematode‐infected trees by leveraging long‐range contextual feature information. Regarding the missed detection in the blue dashed rectangular area of the image, the YOLOv11‐OC model demonstrates superior detection performance.

In comparison with the detection results in the second row, the proposed YOLOv11‐OC model demonstrates excellent performance in detecting small diseased trees in the yellow elliptical area at the bottom of the image. Compared with the original YOLOv11 model, the YOLOv11‐OC model not only accurately identifies smaller diseased regions but also effectively detects diseased trees in the blue dashed rectangular area that were previously missed due to occlusion by other trees. This improved model shows clear advantages in addressing the challenges of small object detection and occlusion.

In summary, this paper improves the C3K2 module of YOLOv11 by introducing the omni‐dimensional dynamic convolution (ODConv), enabling convolution kernels to possess stronger adaptability across different scales and directions, thereby enhancing the detection accuracy of early‐stage small targets. Additionally, the Context Anchor Attention (CAA) mechanism is integrated into the C2PSA module, allowing the improved model to more effectively capture long‐range contextual information, thus enhancing its ability to identify diseased regions of pine wilt nematode‐infected trees. Notably, the improved model shows significant performance gains in detecting diseased tree targets in areas previously missed in the images.

### 3.6 Comparative Experiment on Public Datasets

**FIGURE 9:**
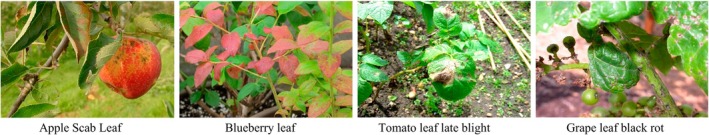
Image samples from the PlantDoc dataset.

To verify the generalization ability of the improved YOLOv11‐OC model, experiments were conducted on the publicly available PlantDoc dataset (Singh et al. 2020). The PlantDoc dataset is specifically designed for plant disease detection and contains a total of 2598 images of plant leaves captured in natural scenes. It covers 13 plant categories and includes 27 common disease types (comprising 17 diseased conditions and 10 healthy conditions). Figure 9 shows some representative image samples from the PlantDoc dataset.

The PlantDoc dataset exhibits a high degree of real‐world complexity, with images often containing challenging interference factors such as complex backgrounds, lighting variations, occlusions, blurriness, and noise. Additionally, the training and testing sets in the PlantDoc dataset have a relatively small number of samples and an uneven data distribution. These factors contribute to relatively lower performance of models trained on this dataset across various evaluation metrics.

Experiments were conducted on the PlantDoc dataset using DETR (Carion et al. 2020), YOLOR‐Light‐v1 (Huang et al. 2023), Model from Lee and Ahn (2023), YOLOv5, YOLOv8, YOLOv11, and the proposed YOLOv11‐OC model. The experimental results are shown in Table 6.

**TABLE 6:** Comparison table of different detection models on the PlantDoc dataset.

Method	F1 score%	mAP50%	mAP50‐95%
DETR (Carion et al. 2020)	—	48.9	46.3
YOLOR‐Light‐v1 (Huang et al. 2023)	—	42.0	32.7
Model from Lee and Ahn (2023)	—	48.2	33.3
YOLOv5	55.2	57.9	44.5
YOLOv8	55.3	57.8	46.0
YOLO11	55.6	59.5	**47.0**
Proposed	**57.3**	**59.9**	46.6

*Note:* Bold values indicate the positions where the corresponding indicators reach their maximum values.

Experiments show that the proposed YOLOv11‐OC model demonstrates outstanding overall performance on the PlantDoc dataset, outperforming other existing methods in both F1 score and mAP50. The F1 score, which considers both precision and recall, indicates that this model ensures high detection accuracy while also exhibiting strong recall ability, resulting in more robust detection performance. It also achieves the best performance in mAP50, reflecting its superior ability to correctly localize targets. Comparative experiments confirm that the model has strong generalization capability for plant disease detection tasks in complex natural scenes.

## 4 Conclusions

This paper proposes a detection method of Pine Wilt Disease based on improved YOLOv11 with UAV remote sensing images. The method optimizes the C3K2 module by introducing the ODConv module into the YOLOv11 architecture, enhancing the model's ability to extract features from small targets and improving the accuracy of detecting early‐stage infected tree areas. Additionally, the method incorporates the CAA attention mechanism to improve the C2PSA module, enhancing the model's contextual awareness and effectively reducing false negatives, thereby improving detection performance in complex backgrounds.

Experimental results show that the proposed YOLOv11‐OC model demonstrates significant advantages in the detection of PWD‐infected trees. The model achieves precision, recall, F1 score, and mAP50 of 94.2%, 83.1%, 88.3%, and 88.9%, respectively, on the PWD tree dataset. Compared to the original YOLOv11 model, the precision, F1 score, and mAP have improved by 2.8%, 1.0%, and 1.4%, respectively. This further validates its effectiveness in detecting PWD infections. Additionally, YOLOv11‐OC also achieved good detection performance on the public PlantDoc dataset, demonstrating its strong cross‐domain generalization ability. In remote sensing image detection tasks, YOLOv11‐OC performs stably and meets practical application needs, providing reliable technical support for the intelligent monitoring of PWD.

Future research will focus on expanding datasets and improving high‐quality annotations, especially as accurately labeling PWD‐infected trees in drone remote sensing imagery remains a significant challenge. Additionally, more advanced data augmentation techniques will be explored to integrate feature information from multiple data sources, thereby enhancing the model's robustness and generalization capabilities.

## Author Contributions

**Hua Shi:** conceptualization (equal), funding acquisition (equal), methodology (equal), writing – review and editing (equal). **Zhenhui Zhu:** methodology (equal), writing – original draft (equal). **Xiaozhou Feng:** conceptualization (equal), validation (equal). **Yufen Xie:** validation (equal). **Hui Guo:** validation (equal). **Pengxiang Xue:** data curation (equal), software (equal). **Yonghang Wang:** software (equal).

## Funding

This work was supported by Xi'an Technological University through two projects: the PWD Image Detection Model Research Project Based on Machine Learning Methods (Grant No. H20247273) and the Xi'an Technological University Graduate Education and Teaching Reform Research Project (Grant No. XAGDYJ240107).

## Conflicts of Interest

The authors declare no conflicts of interest.

## Data Availability

The data presented in this study are available on request from the corresponding author.
